# Increasing functional modularity with residence time in the co-distribution of native and introduced vascular plants

**DOI:** 10.1038/ncomms3454

**Published:** 2013-09-18

**Authors:** Cang Hui, David M. Richardson, Petr Pyšek, Johannes J. Le Roux, Tomáš Kučera, Vojtěch Jarošík

**Affiliations:** 1 Centre for Invasion Biology, Department of Botany and Zoology, Stellenbosch University, Matieland 7602, South Africa; 2 Institute of Botany, Department of Invasion Ecology, Academy of Sciences of the Czech Republic, CZ-252 43 Průhonice, Czech Republic; 3 Department of Ecology, Faculty of Science, Charles University in Prague, Viničná 7, CZ-128 44 Praha 2, Czech Republic; 4 Department of Ecosystem Biology, Faculty of Science, University of South Bohemia, Branišovská 31, CZ-370 05 České Budějovice, Czech Republic

## Abstract

Species gain membership of regional assemblages by passing through multiple ecological and environmental filters. To capture the potential trajectory of structural changes in regional meta-communities driven by biological invasions, one can categorize species pools into assemblages of different residence times. Older assemblages, having passed through more environmental filters, should become more functionally ordered and structured. Here we calculate the level of compartmentalization (modularity) for three different-aged assemblages (neophytes, introduced after 1500 AD; archaeophytes, introduced before 1500 AD, and natives), including 2,054 species of vascular plants in 302 reserves in central Europe. Older assemblages are more compartmentalized than younger ones, with species composition, phylogenetic structure and habitat characteristics of the modules becoming increasingly distinctive. This sheds light on two mechanisms of how alien species are functionally incorporated into regional species pools: the settling-down hypothesis of diminishing stochasticity with residence time, and the niche-mosaic hypothesis of inlaid neutral modules in regional meta-communities.

## Abstract (web summary)

Understanding how species assemblages change over time is crucial for conservation. Here, the authors assess the changes of compartmentalized structure in native and alien species across millennia, and show that older assemblages can form more functionally distinctive modules than younger ones.

## Graphical abstract

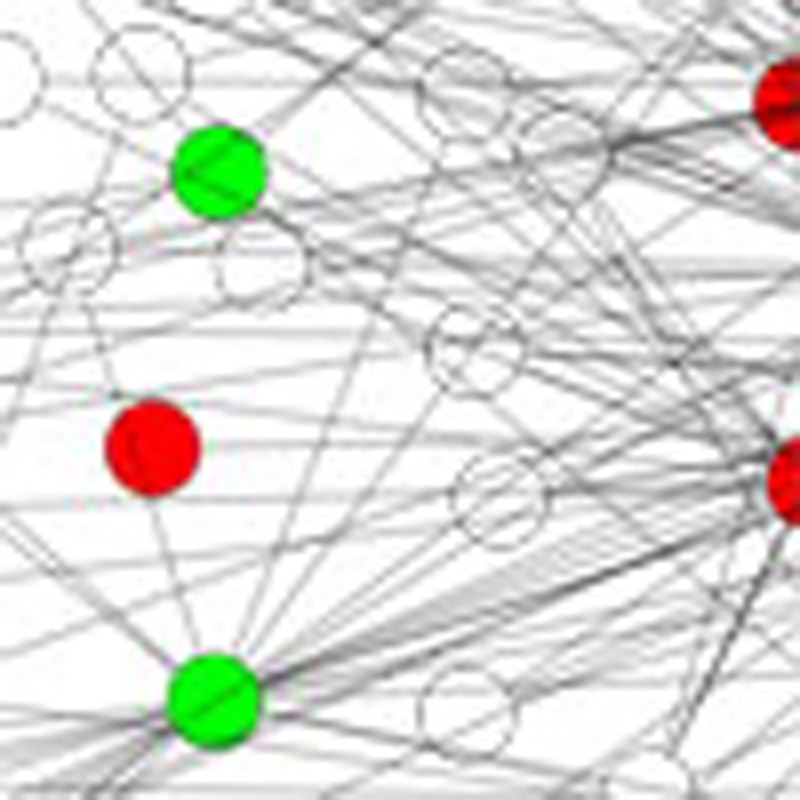

Ecological processes, environmental filters and stochasticity are constantly driving the compositional and structural changes of species co-distribution at local and regional scales^1^. Knowing the trajectories of these changes is central to ecology and crucial for efficient conservation management^2^. In local communities, resource competition and cross-trophic interactions after disturbance are the main drivers of structural changes^3^,^4^. In regional meta-communities, environmental filtering and dispersal limitation are thought to mediate the formation of species assemblages^5^,^6^, yet these two processes are constantly disrupted by human-driven forces, leading to the current phase of biotic homogenization^7^. Despite the urgent need to better quantify and interpret these compositional and structural changes at regional scales, identifying appropriate long-term data (for example, paleobotanical records) and sensitive indicators of structural changes remains challenging.

Biological invasions create an ideal experiment for elucidating the potential trajectories of regional changes in species co-distribution. Introduced species need to cross a series of filters to become naturalized and invasive, forming an introduction–naturalization–invasion continuum, hereafter INIC^5^,^8^. The stochastic component of ‘random’ introduction is gradually diminished through multiple dispersal and environmental filters, with the remnant species emerging as ‘winners’. These filters thus define the direction in both human-mediated and natural selections—towards better performance in novel environments^5^,^8^. Categorizing species at the same trophic level according to their residence time into regional assemblages of different ages and then examining the structural differences between these assemblages may capture the signal of the regional structural changes^9^. Although these species with different residence times do interact, the role of interspecific interactions within a single trophic level at the regional scales is relatively trivial compared with top–down regional processes—driven specifically by habitat suitability and dispersal barriers—in regulating locally unsaturated assemblages^3^,^6^,^10^. Consequently, the co-distribution of species in multiple sites resembles a bipartite resource–consumer network (for example, a host–parasitoid network), with species as consumers and sites as resource providers.

We derive two specific hypotheses to unveil the potential trajectories of compositional and structural changes in regional assemblages along the INIC. First, as species in older assemblages are winners and survivors of longer selection, stronger signals of matching between their habitat requirements and the characteristics of inhabited sites should be expected (that is, a lock-and-key relationship), with groups of species likely to inhabit non-random subsets of sites that reflect this match. In other words, species and sites in older assemblages are expected to belong to largely disjoint modules (or communities), and should thus show a compartmentalized structure. In contrast, more recent introductions should have a poorer match as many species are initially randomly introduced to sub-optimal sites. At the regional level, we would thus expect to see a higher level of compartmentalization (that is, modularity) in older assemblages (hypothesis I: the settling-down hypothesis of diminishing effect of stochasticity with residence time). Modularity analyses, also known as community detection, have often been employed to better understand the topography and stability of food webs^11^,^12^,^13^,^14^,^15^. Given a network with nodes connected by edges, we need to identify specific ways of partitioning nodes into non-predefined non-overlapping groups so that the number of within-group connections relative to random expectation is maximized (that is, like is connected to like in a network^16^). To the best of our knowledge, this is the first attempt to utilize modularity to quantify structural changes in species assemblages resulting from biological invasions.

Second, the importance of neutral versus niche-based processes in shaping species assemblages has been fiercely contested^17^,^18^,^19^. Species in neutral assemblages are considered ecologically identical^10^, and thus species composition, evolutionary divergence and habitat characteristics of different modules, if present, should be indistinguishable; this should result in assemblages compiled through stochastic factors. In contrast, species in niche-based assemblages have different functional roles^4^,^20^,^21^, leading to modules with distinct taxonomic composition, evolutionary units and habitat characteristics, reflecting a deterministically (or functionally) driven species assemblage^22^. Theoretically, biodiversity maintenance and species coexistence can be achieved by being either ecologically identical or distinctive^23^, forming niche-differentiated modules (or communities) that comprise species with rather similar niche within a module^24^. We thus expect that the modules will become more functionally distinctive with an increase in residence time; that is, the shift from an initially neutral or stochastic assemblage to a niche-based functional-driven multi-module assemblage along the INIC (hypothesis II: the niche-mosaic hypothesis of inlaid neutral modules in the regional meta-community).

To test these two hypotheses, here we categorize all recorded vascular plant species in the network of nature reserves in the Czech Republic, central Europe^25^, as natives (present in the region since the last glaciation), archaeophytes (historical immigrants that were introduced to Europe between the initiation of agricultural activities during the Neolithic period (ca. 4000 BC) and the European exploration of the Americas (ca. 1500 AD)) and neophytes (modern invaders introduced into Europe after 1500 AD)^26^. Archaeophytes, having been present for several millennia in central Europe, represent the transition between native species and neophytes in terms of invasion dynamics, habitat affiliations and interaction with other trophic levels^9^,^27^,^28^,^29^. Comparisons of modularity are made for these alien and native assemblages representing different residence times. This extraordinary data set enables us to amplify the signals of structural changes in regional assemblages that are often weak or unidentifiable in studies conducted over a short period.

## Results

### Modularity of assemblages

The data set comprised 2,054 species from 135 families in 302 reserves in the Czech Republic, with 4 families contributing ≥5% of the total number of species: Asteraceae 14.8%, Poaceae 7.8%, Rosaceae 5.7% and Cyperaceae 5.2%. The list contained 1,686 native taxa from 122 families, 212 archaeophyte taxa from 37 families and 156 neophyte taxa from 48 families. All these vascular plant species, native or introduced, formed their current assemblages through colonization after the last glaciation, with many of them present as invaders in other parts of the world (Supplementary Note 1).

**Figure 1:**
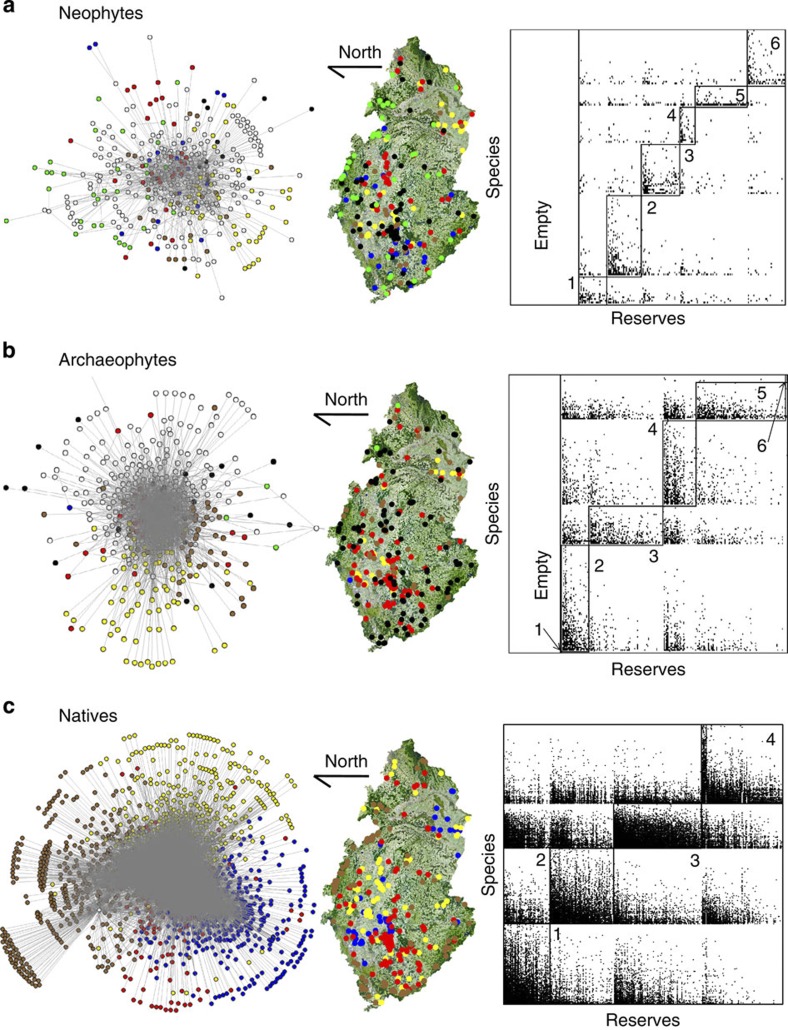
Network structures of vascular plants in the Czech Republic. Network expression, geographical location of reserves and species-by-site matrix of modules identified for (**a**) neophytes, (**b**) archaeophytes and (**c**) natives. In the network expression, open circles represent reserves. Blue, yellow, red, brown, black and green points in the network expression and geographical maps indicate different modules identified in each of the three assemblages. Modules in the matrices are marked by the serial numbers and a rectangle, with points indicating the presence of a species (a row) occurring in a reserve (a column) and the rectangles of ‘Empty’ in neophytes and archaeophytes indicating reserves where these two species assemblages do not occur.

**Figure 2:**
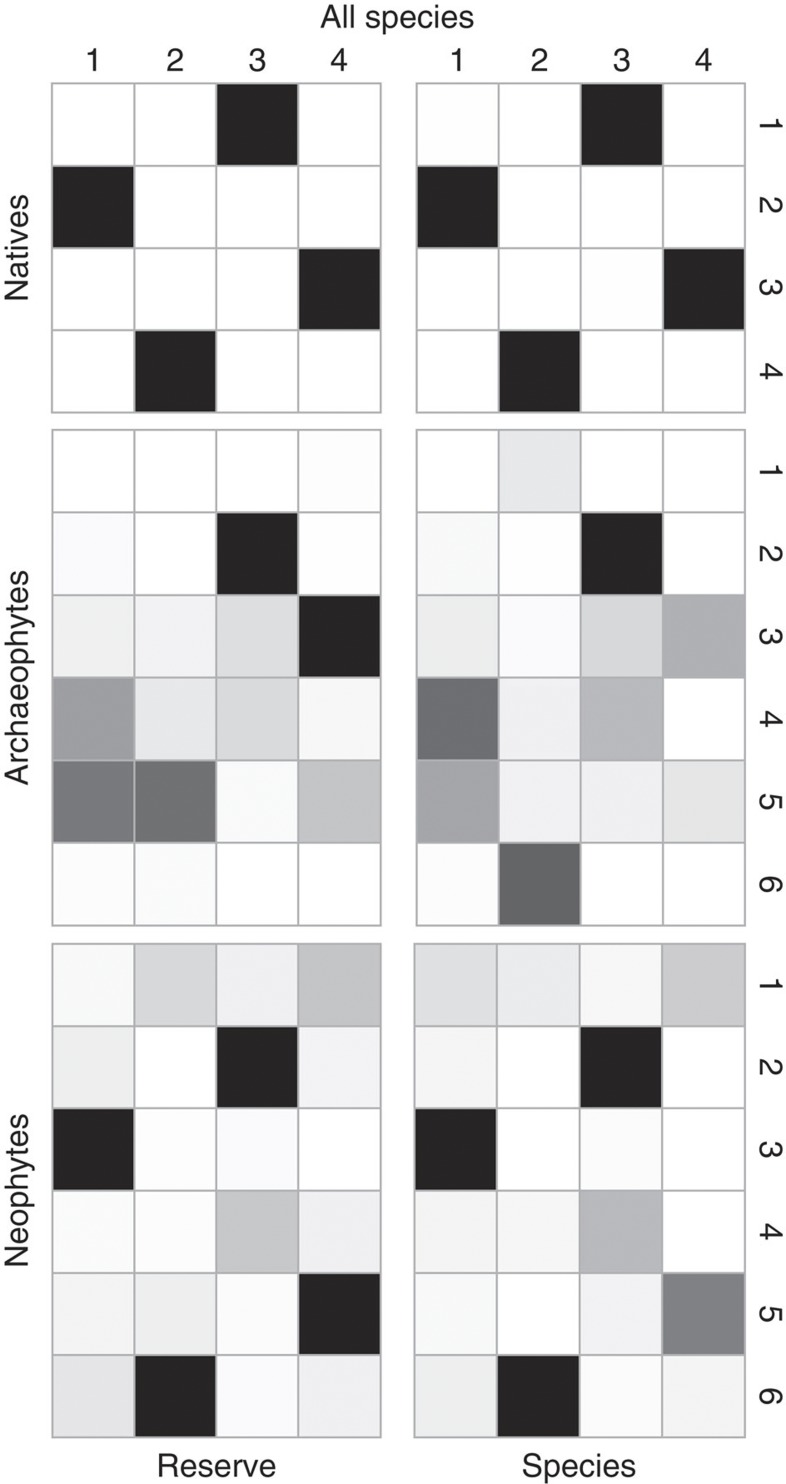
The Jaccard similarity between modules of the entire assemblage and separated assemblages. Black indicates completely similar (*J*=1), and white completely non-overlapping (*J*=0) of species or reserves (see Supplementary Note 2 for details).

All three assemblages were significantly compartmentalized (neophytes: 6 modules, *Z*-test, *M*_*z*_=7.98, *P*<0.01; archaeophytes: 6 modules, *Z*-test, *M*_*z*_=15.94, *P*<0.01; natives: 4 modules, *Z*-test, *M*_*z*_=175.65, *P*<0.01), with the modules identified as being visible when viewed as network diagrams, geographical sites and species-by-reserve matrices (Fig. 1). Modules identified separately for these three assemblages are largely consistent with those identified for the combined assemblage of all species and reserves (Supplementary Note 2), indicating a roughly one-to-one matching (4 modules, *Z*-test, *M*_*Z*_=163.61, *P*<0.01; Fig. 2), with the within-module degree significantly differing for assemblages and modules (Supplementary Table S1 and Supplementary Fig. S1).

The intensity of compartmentalization increasingly deviates from the null model expectation (that is, the increase of *M*_*z*_) as we move from young to mature assemblages along the INIC (that is, from neophytes to archaeophytes and then to natives). Adding a random assemblage generated from the null model (thus with *M*_*z*_<1.96), we can then see a perfect trend (Spearman’s rank correlation *ρ*=1.0, *P*<0.05), supporting hypothesis I that assemblages compiled according to residence time become more compartmentalized along the INIC.

**Figure 3:**
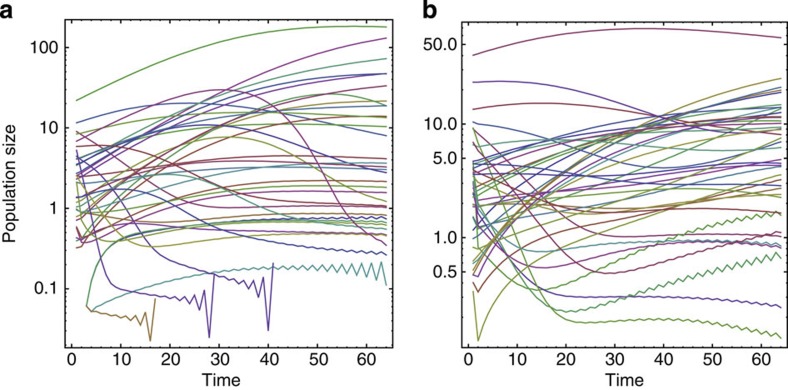
Population dynamics of the Lotka–Volterrra model. (**a**) Population dynamics of different species in a single site; (**b**) population dynamics of a single species in different sites. Note that the population size is log-transformed (see Supplementary Note 3 for details).

**Figure 4:**
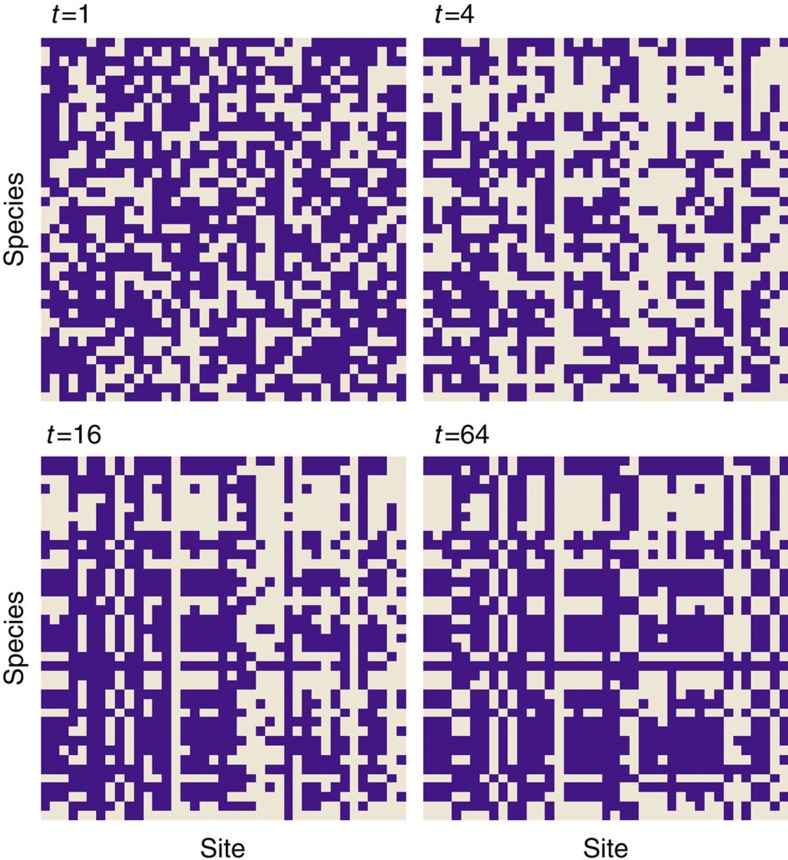
The dynamics of species-by-site matrix in the Lotka–Volterra model. A cell with dark colour indicates the species on the same row occurs in the site of the same column (see Supplementary Note 3 for details).

Simulations using the Lotka–Volterra model of meta-communities (Supplementary Note 3) also supported a rising modularity with time. Specifically, the dynamics of population size vary dramatically, and a suite of uniquely combined species gradually settle down and persist in specific sites (Fig. 3). In contrast to the rather chaotic population dynamics, the network structure as depicted by the species-by-site matrix showed a steady trend from randomness to more compartmentalized structures (Fig. 4). Furthermore, the standard modularity *M*_*Z*_ of subset assemblages behave rather similarly to the entire assemblage (Supplementary Fig. S2), supporting that the assemblage-for-time substitution of categorizing species in a regional pool into subsets of different residence times is theoretically valid.

### Functional distinctiveness of modules

**Figure 5:**
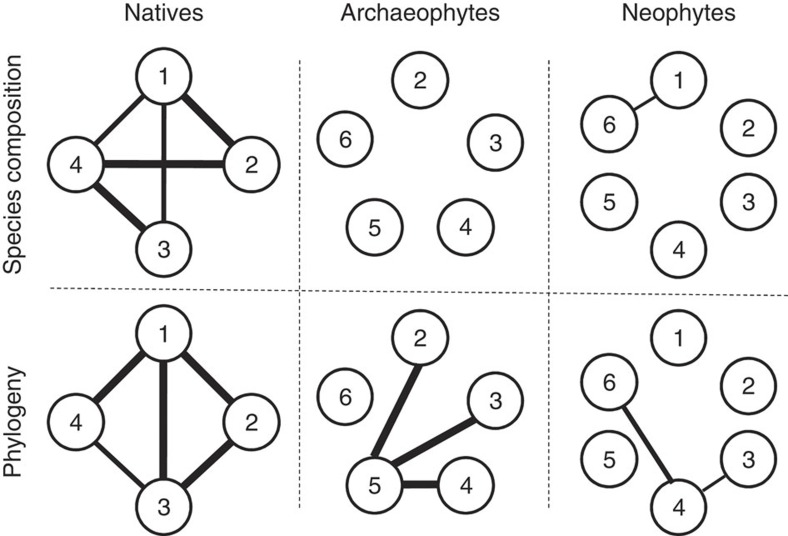
Relationships between modules of species composition and phylogenetic divergence. Solid lines represent significant differences between the two linked modules, with the thickest, intermediate and thinnest lines indicating *P*-values of <0.001, <0.01 and <0.05 (KS test for species composition and Kruskal–Wallis test for phylogenetic divergence), respectively (see Supplementary Data 1 and 2 for details).

Modules become more distinctive in older assemblages (Fig. 5) in terms of both species composition (that is, the number of species in each family; see Supplementary Data 1) and phylogenetic relatedness (see Supplementary Data 2). Specifically, except for module 2 and 3 (*D*_*F*_=0.97, *P*>0.05), the Kolmogorov–Smirnov test showed that all other pairwise modules of natives (five out of six) are significantly different from each other (*D*_*F*_>1.71, *P*<0.01). Except for modules 2 and 4 (*P*=0.13), Kruskal–Wallis tests also indicated that between-module phylogenetic distances of natives (five out of six) are significantly greater than within-module distances (*P*<0.01). In contrast, only 1 out of 15 possible pairs of neophyte modules is compositionally distinctive (Fig. 5), and only 3 out of 10 possible pairs of archaeophytes modules and 2 out of 15 possible pairs of neophyte modules are phylogenetically distinctive (Fig. 5). This supports hypothesis II that modules within assemblages become more distinctive along the INIC.

**Figure 6:**
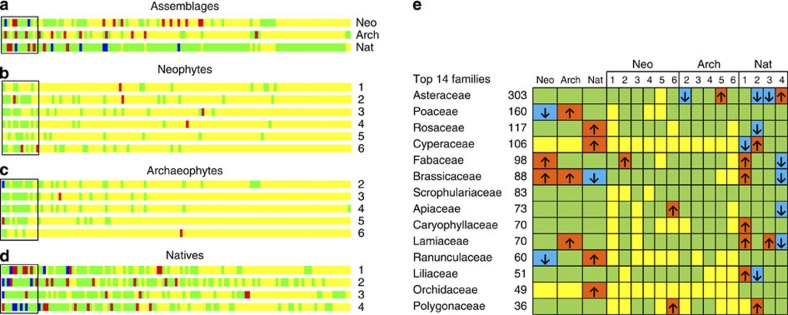
Fingerprints of species composition in modules and assemblages. The species compositions of 135 families are compared with random draws from 10,000 simulations (**a**–**d**). Families are sorted according to the number of species in the total assemblage and arranged from the left to right. Yellow, green, red and blue bars indicate the families that are absent, present, over- and under-represented, respectively. Details of the top 14 species-rich families are given in (**e**). Up-facing and down-facing arrows indicate over- and under-representation respectively (also see Supplementary Note 1).

Comparisons between modules and assemblages revealed fingerprints of over- and under-representing certain families (Fig. 6; also see Supplementary Note 1). Before 1500 AD, families of true grasses (Poaceae), mustards (Brassicaceae) and mints (Lamiaceae) were overrepresented in plant introductions (Fig. 6a,e). In contrast, legumes (Fabaceae) and mustards were overrepresented, while families of true grasses and buttercups (Ranunculaceae) were underrepresented among neophytes (Fig. 6a), indicating fewer introductions of true grasses after 1500 AD. Modules of neophytes showed no obvious contrasts (Fig. 6b,e) but only overrepresented legumes in module 2 and carrots (Apiaceae) and knotweeds (Polygonaceae) in module 6. Modules of archaeophytes indicated one contrast (that is, under- versus over-representation) between modules 2 and 5 for the daisy family (Asteraceae) (Fig. 6c,e). Comparisons between modules of natives showed more contrasts between modules for families of daisies, sedges (Cyperaceae), legumes, mustards, mints and lilies (Liliaceae) (Fig. 6d,e), supporting hypothesis II that there are more functional contrasts between modules with residence time.

### Habitat differentiation between modules

**Figure 7:**
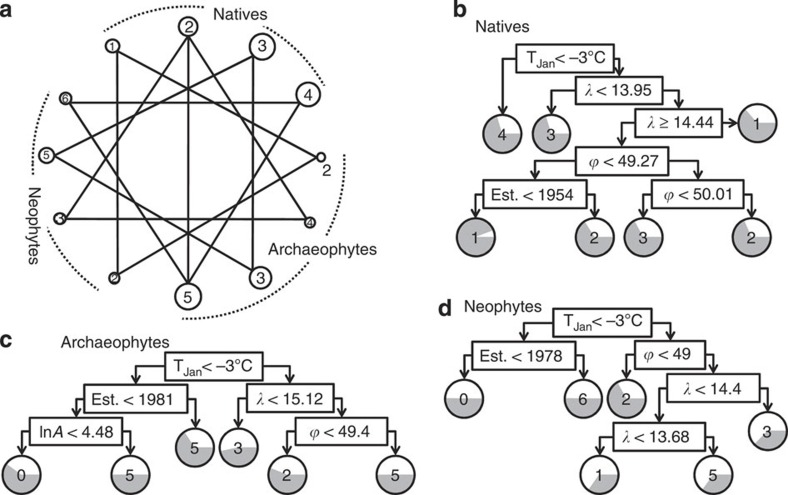
Habitat differentiation and characteristics of modules. (**a**) Geographical overlaps between modules of reserves, with a solid line indicating a substantial similarity (Jaccard’s similarity *J*>0.2). (**b**–**d**) Classification trees after pruning using seven habitat characteristics, including log-transformed reserve size (ln*A*), number of habitat types, year established (Est.), longitude (*λ*), latitude (*ϕ*), average temperature in January (*T*_Jan_) and human density, to predict the membership of reserve modules. Grey pie chart in the module circles indicates the successful rate of predicting specific modules.

Modules of reserve composition are geographically consistent across different assemblages (see the triangular edges in Fig. 7a), with each module in an assemblage overlapping spatially with a specific module from another assemblage (Jaccard’s similarity *J*≥0.2). After removing variables with strong collinearity from the 14 habitat descriptors and two outliers of old reserves (Boubínský and Hojná voda primeval forests; *Z*-test, Mahalanobis distances *D*_*ij*_^*2*^>31.8, *P*<0.001), the classification tree of the remaining seven variables (log[reserve size], habitat diversity, year of establishment, longitude, latitude, average temperature in January and human density, with VIF<2) showed that the between-module habitat differences were significant for all three assemblages (Wilks’ *λ*>0.28, *P*<0.001). Misclassification error rates from pruning the classification tree (with the complexity parameter *cp*=0.02) were low for natives (22.7%; Fig. 7b) and moderate for archaeophytes (45%; Fig. 7c) and neophytes (43.3%; Fig. 7d), but still much lower than the error rates for randomly assigning reserves to modules (3/4, 6/7 and 6/7 for natives, archaeophytes and neophytes, respectively).

Overall, vascular plant species with close phylogenetic relatedness in central Europe form modules that show signals of over- and under-representing specific families (Fig. 6), and species of the same module are likely to co-occur in a group of reserves with certain criteria of winter temperature, year of establishment and spatial locations (Fig. 7), reflecting the lock-and-key relationship between their habitat requirement and the characteristics of the inhabiting reserves. Combining Figs 6 and 7 yields a better understanding of this lock-and-key relationship, with important management implications. For instance, native daisies prefer reserves with cold winters (*T*_Jan_<−3 °C), whereas native legumes, mustards and mints prefer the western parts of the country (λ<13.95) with relatively warmer winters (*T*_Jan_>−3 °C). Reserves with cold winters (*T*_Jan_<−3 °C) and older establishment (pre 1980) seem to resist the invasion of archaeophytes and neophytes.

## Discussion

As regional ecosystems are open-ended and constantly evolving systems^30^, their changes should be better reflected by system structures and orders. Although many other structural indices, especially nestedness, have been proposed to capture structural and functional changes in species-by-site matrices of co-distribution^31^ and bipartite ecological networks^32^, there has been no consensus on whether nested structure enhances resilience against perturbation^33^,^34^,^35^ or weakens species persistence^36^,^37^. In this regard, compartmentalization, although partially related to nestedness^38^, has been shown to increase network stability^39^,^40^. Moreover, such an approach allows posterior between-module comparisons that can yield crucial knowledge directly linked to efficient conservation planning and management. Specifically, the identified classification criteria for reserve modules (and the similar kind for species modules once quantitative descriptors of species are available) provide a powerful tool for connecting the invasibility of site with the invasiveness of species. This is attractive to invasion science because, historically, lock-and-key relationships were explored separately^41^. The increasingly availability of data sets on the life-history traits of alien species and site characteristics now makes it feasible to examine interactions between these factors.

The role of species’ traits and niche functions in structuring species assemblages has been hotly debated^20^,^21^,^42^, largely due to the strong dichotomy between neutral-stochastic and niche-based models. Placing the genesis of ecosystems into one of these categories is often done by comparing the similarity of assemblage patterns generated from these models with real-life observations. As different processes can lead to similar patterns, such pattern comparisons cannot provide conclusive support for the mechanisms embedded in the model^43^. Our approach of assessing species composition and phylogeny in and between modules of different age classes takes us a step beyond examining only the co-distribution patterns of species associations. Modules comprising neophytes showed little differentiation, in contrast to the high distinctiveness of modules comprising natives, supporting the transition from neutral-stochastic processes to niche/functional-based processes in governing the regional meta-communities. With the increase of residence time along the INIC^9^, environmental filters drive a largely randomly assembled species of neophytes to a regional assemblage of natives with unique functional clusters (that is, the niche-mosaic hypothesis), consistent with the theoretical prediction by Scheffer and van Nes^24^. Our results suggest the decreasing role of stochastic forces and the increasing importance of deterministic processes as species move along the INIC^9^,^22^. This increasing role of deterministic processes relative to the diminishing role of stochasticity in assembling regional species lists (that is, the settling-down hypothesis) emphasizes the long-term structural changes in meta-communities^44^,^45^ and offers a temporal perspective for reconciling the debate between neutral and niche-based schools of thought.

We need to highlight that the analysis of modularity here was based solely on the species-by-reserve matrices, without using any information on the kind of species or the habitat characteristics of these reserves. The analysis of modularity thus provides an opportunity for posterior examination and comparison of detected sub-communities and modules that can potentially expose how systems assemble and function, as supported here by the settling-down hypothesis I and the niche-mosaic hypothesis II. Refined conservation plans could be designed for each module. This module-based risk assessment and planning are consistent with the trait- and function-based conservation and deserve further analyses for other regions. As these matrices only depict the co-distribution pattern of species association, our results from the posterior analyses of identified modules thus suggest that species co-distribution could be more informative than species distribution for quantifying species invasiveness and performance in novel environments^46^, non-random species associations emerge along the INIC, and these co-distribution pattern of species association reflect the match between species’ functional roles and their habitat requirement, supporting hypotheses I and II. Categorizing species into different assemblages according to their residence time along the INIC provides a method for exploring the structural changes caused by biological invasions. The increasing modularity from young to mature assemblages not only identifies a specific facet of the directional change in regional assemblages but also suggests a transition from an assemblage driven by stochastic process to functional-driven multi-module assemblages along the invasion pathway of INIC.

## Methods

### Species categorization

Lists of vascular plant species for reserves in the Czech Republic were collected and updated from published records and floristic inventories at the Agency for Nature Conservation and Landscape Protection, Prague^25^. Archaeophytes are defined as plant species that were intentionally or unintentionally introduced into Europe between the initiation of agricultural activities during the Neolithic period (ca. 4000 BC) and the European exploration of the Americas (ca. 1500 AD), respectively^26^. Plant species introduced into Europe after 1500 AD were classified as neophytes. The two groups differ in their invasion characteristics and ecology due to the contrasting regimes of selection and cultivation operating in ancient and modern societies^26^. Most archaeophytes originated from southern Europe and most are associated with dry habitats, grasslands and agricultural landscapes, whereas most neophytes originated from outside Europe and are common in warm areas, where they invade different habitats on both dry and wet sites^27^. The separation between natives and archaeophytes in regional floras relies on a combination of paleobotanical, archaeological, ecological and historical evidence^9^.

### Level of compartmentalization

To test whether older assemblages are more compartmentalized than younger assemblages (hypothesis I: a settling-down process of diminishing stochasticity), we compared the modularity of the three assemblages. The modularity (*M*) of a species-by-site matrix is calculated by maximizing Newman and Girvan’s^47^ definition of compartmentalization through partitioning species and sites into modules. To solve the potential resolution problems^48^ and the sensitivity to the initial situation and ending criteria, we used the simulated annealing in the Netcarto programme^49^,^50^, with both species and reserves treated as network nodes. Although other approaches exist for bipartite networks, we here chose Netcarto because it has good performance for bipartite networks^51^ and further allows for connecting species traits with reserve characteristics within a single module (that is, the lock-and-key relationship). We used the *Z*-score of modularity for comparing across assemblages, *M*_Z_=(*M−M*_*N*_)/SD_*N*_, where *M*_*N*_ and SD_*N*_ are the average and standard deviation of modularity from 1,000 random matrices with the same ranking of node degrees as the observed matrices^50^.

To support the contention that biotic interactions between natives, archaeophytes and neophytes have trivial effects on the modularity analysis at the regional scale^3^,^6^, we calculated the modularity for the combined assemblage of all species and reserves using the same method (Supplementary Note 2). Once modules were identified for the combined assemblage, we then calculated the within-module degree and participation coefficient for each species^52^. These two coefficients depict how the node in a network is positioned in its own module and with respect to other modules^53^,^54^. We also conducted an analysis of variance for both within-module degree and participation coefficient, with assemblages and modules as factor variables.

### Lotka–Volterra model

To justify the substitution of temporal assemblage changes by comparisons of subset assemblages with different residence times, we need to support three prerequisites of the assemblage-for-time substitution (Supplementary Fig. S3). First, the modules identified for the subset assemblages are consistent with those detected for the entire assemblage (Supplementary Note 2). Second, the modularity dynamics of a subset assemblage is correlated (synchronized) with that of the entire assemblage. Third, the modularity of the entire species assemblage increases temporally in a meta-community with competitive species in multiple interconnected sites. To support the last two prerequisites of the assemblage-for-time substitution, we built a widely applied Lotka–Volterra model (Supplementary Note 3); this mathematical model depicts competitive coexistence of multiple species in multiple sets connected by dispersal (Supplementary Note 3). We then recorded the dynamics of population size, species-by-site matrix and the modularity of the entire and a subset assemblage (50% of species), reflecting the succession dynamics of species composition and co-distribution network structure in an ecological meta-community.

### Species composition

To test whether modules of older assemblages are functionally more distinctive (hypothesis II: the niche-mosaic structure of inlaid neutral modules in a regional meta-community), we compared the species composition, phylogenetic relatedness and habitat characteristics of each module identified. Specifically, to examine the species composition for each of the three assemblages, we performed a re-sampling of the species without replacement, repeated 10,000 times. Specifically, for each re-sampling we randomly chose an equal number of species to the focal assemblage (or module) from the list of all species (or the resided assemblage) and counted the number of species for each family, from which the confidence intervals of the number of species in each family can be determined and compared with the observed number of species. This provides a fingerprint of which family is over- and under-represented in each assemblage or module. The overall difference of the species composition between two modules (or two assemblages) was examined by using a two-sample Kolmogorov–Smirnov (KS) test (Supplementary Data 1); specifically, we calculated a dimension-free distance between the number of species of each family, *D*_*F*_=*D*_*KS*_(*n*_1_*n*_2_/(*n*_1_+*n*_2_))^1/2^, where *D*_*KS*_ is the KS distance, *n*_1_ and *n*_2_ the number of species of the two modules, and the critical value for rejecting the null hypothesis that the species composition of the two modules are the same is *D*_*F*_>1.36 (KS test, *P*<0.05).

### Phylogenetic signal

To check for signals of phylogenetic divergence (at genus level) within and among modules, we obtained molecular data for the ribulose-bisphosphate carboxylase (rbcL) gene region for representatives of all genera for which data were available in GenBank ( ncbi.nlm.nih.gov; details and accession numbers see Supplementary Data 3 and 4). For those species in our data set with no available data, we randomly chose a closely related species in the same genus where possible. Our final dataset comprised 537 taxa, representing 72% of all genera (959) represented in our species list. Sequence data were aligned and manually edited to a final matrix consisting of 1,407 characters that contained 18 gaps (indels) ranging between 1–31 base pairs. The average number of nucleotide substitutions per site between sequences was calculated using a maximum composite likelihood model implemented in MEGA5 (ref. 55); all ambiguous positions were removed. We compared these genetic distances between all possible species pairs within and between identified modules using the Kruskal–Wallis tests (Supplementary Data 2).

### Habitat characteristics

Nature reserves are aimed at protecting relatively undisturbed natural vegetation, which has a long uninterrupted history in the region, and thus represents an ideal data set for such analyses of habitat differentiation between identified modules. To examine the habitat characteristics of each module, we further compiled a list of 14 environmental descriptors of the reserves, including the year established, number of habitat types (physiotypes), physical feature (longitude, latitude, area size, middle, minimum and maximum elevation, elevation range), climate (annual precipitation, mean annual temperature, average temperature in January and in June) and human density^25^,^56^. To run the classification tree analysis used in Fig. 7, we used the R statistic computing language (version 2.15.1)^57^. Specifically, for the 14 environmental descriptors we first checked the collinearity using the command corvif(·) in the AED package^58^. We sequentially removed the variable with the highest variance inflation factor (VIF) and then re-ran the command corvif(·) until the VIFs of all remaining variables were <2.0; this procedure gave us a list of seven variables, including log-transformed area sizes, number of habitat types, established time, longitude, latitude, temperature in January and human density. Using modules as the dependent variable, we ran the recursive partitioning using the command rpart(·) in the rpart package^59^. For the generated trees, we ran a cross-validation using plotcp(·) to decide a reasonable complex parameter and then pruned these trees using the command prune(·) with the specific complexity parameter (*cp*=0.02) identified during the cross-validation.

## Author contributions

C.H. and D.M.R. conceptualized the research; C.H., P.P., J.J.L.R., T.K., and V.J. prepared and analysed data; and C.H., D.M.R., P.P. and J.J.L.R. wrote the paper.

## Additional information

**How to cite this article:** Hui, C. *et al.* Increasing functional modularity with residence time in the co-distribution of native and introduced vascular plants. *Nat. Commun.* 4:2454 doi: 10.1038/ncomms3454 (2013).

## Supplementary Material

Supplementary Figures, Supplementary Table, Supplementary Notes and Supplementary References Supplementary Figures S1-S3, Supplementary Table S1, Supplementary Notes 1-3 and Supplementary References

Supplementary Data 1 Dimension-free Kolmogorov-Smirnov distances

Supplementary Data 2 Significance of the Kruskal-Wallis test for phylogenetic distances

Supplementary Data 3 Genus-level phylogeny

Supplementary Data 4 Accession numbers of GenBank for building the phylogenetic distance matrix
