# Supplementary material for: Increasing functional modularity with residence time in the co-distribution of native and introduced vascular plants
Source: Nat Commun. 2013 Sep 18;4:2454. doi: 10.1038/ncomms3454 (PMC3791474; doi:10.1038/ncomms3454)
Supplement: Supplementary Figures, Supplementary Table, Supplementary Notes and Supplementary References — Supplementary Figures S1-S3, Supplementary Table S1, Supplementary Notes 1-3 and Supplementary References [file ncomms3454-s1.pdf]

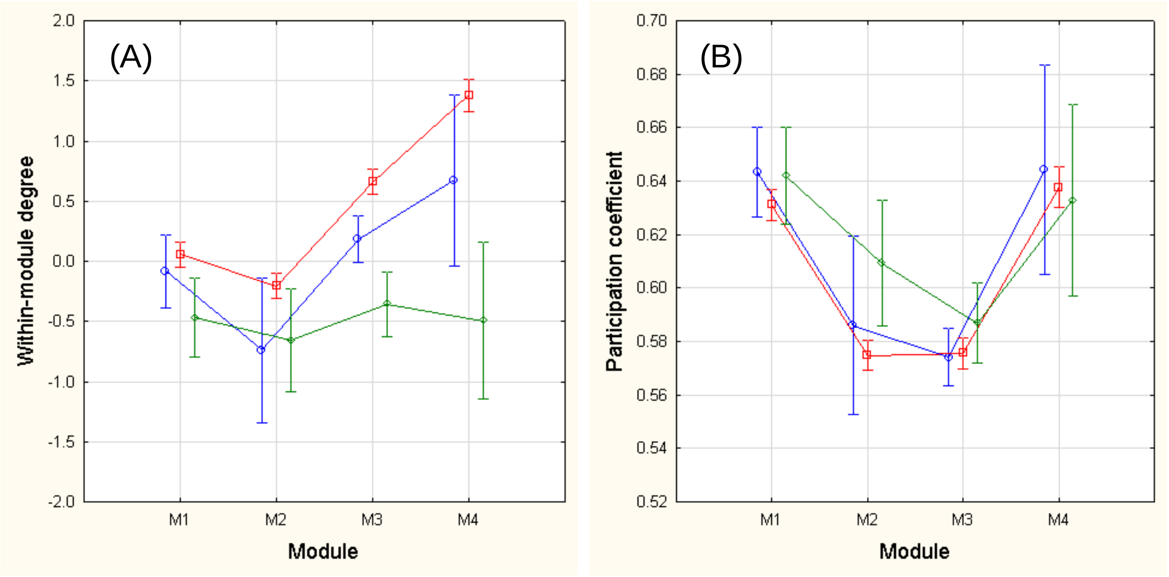

Supplementary Figure S1. The role of species in the co-distribution network. Within-module degree (A) and participation coefficient (B) of the four modules identified for natives (red), archaeophytes (blue) and neophytes (green) in the combined assemblage. Details of the number of species and standard error for each module see Supplementary Table S1.

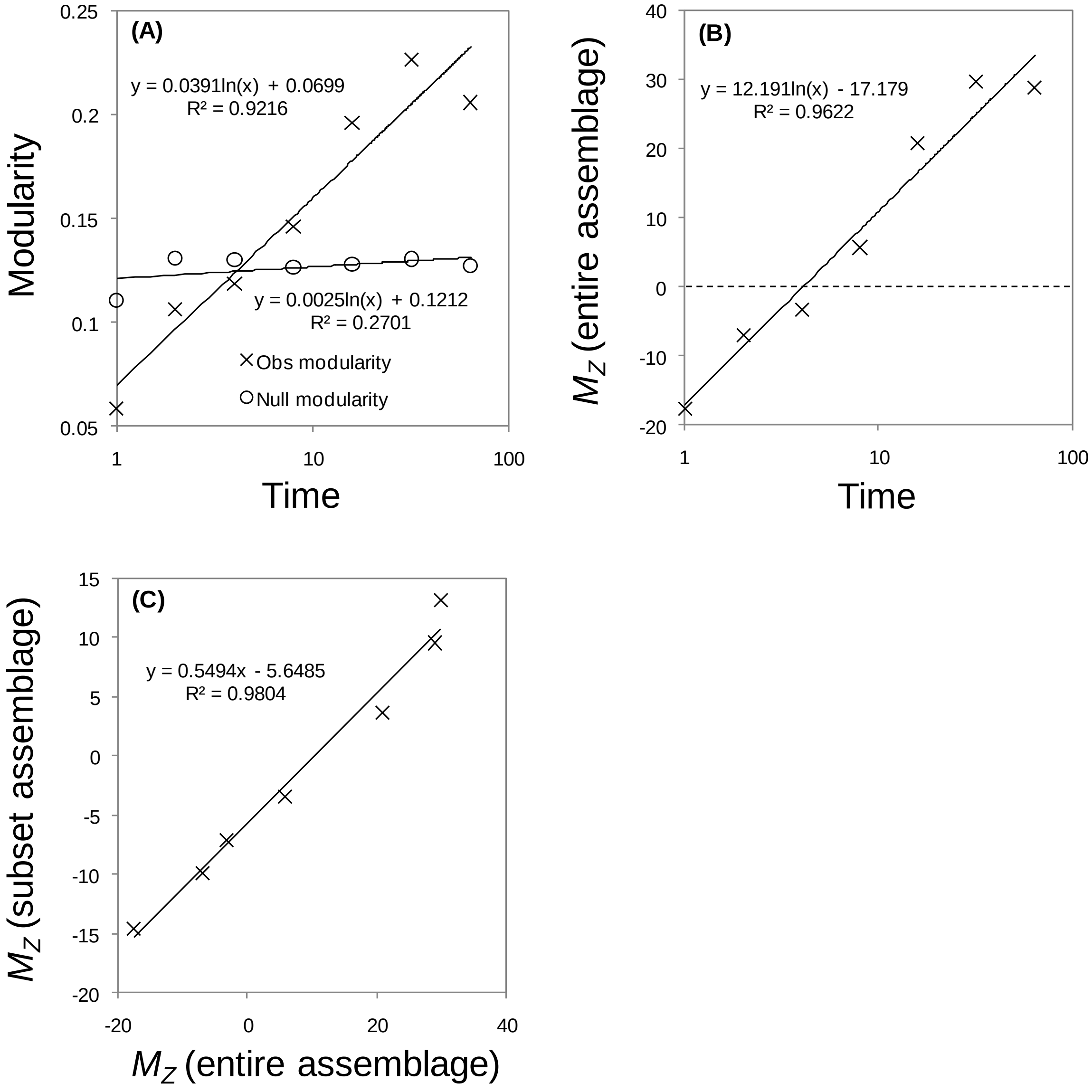

Supplementary Figure S2. The dynamics of modularity in the simulation. (A) The dynamics of absolution modularity; (B) the dynamics of standardized modularity; (C) the relationship between the standardized modularity for the entire and a subset assemblage.

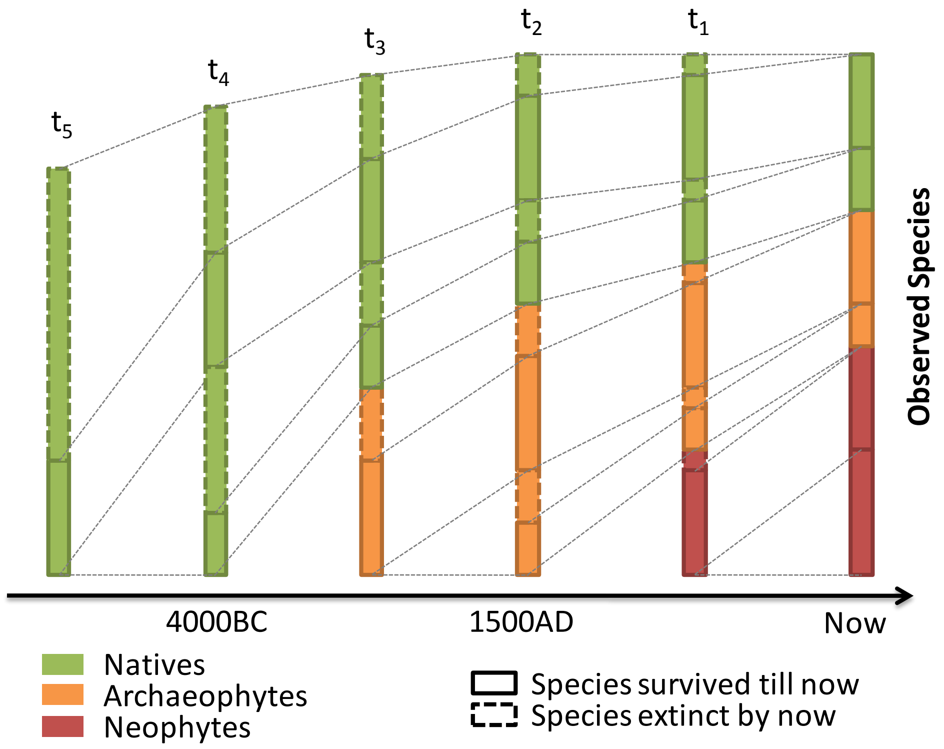

Supplementary Figure S3. An illustration of potential temporal dynamics of species assemblages. The regional species assemblages are dynamic. Current assemblages (the rightmost bar) comprise species that have survived until now, whereas historical snapshots remove species that will go extinct over time and those added through introduction events. The problem of comparing the current native assemblage (the rightmost green bar) with natives plus archaeophytes (the rightmost green and orange bars) is not the same as comparing the assemblages at t_4 with those at t_3 because different numbers of natives and archaeophytes have been lost and new species will still be added into the archaeophytes.

Supplementary Table S1. A summary of within-module degree (z_i) and participation coefficient (P_i).

Assemblage	Module	# species	z_i_Mean	z_i_S.E.	P_i_Mean	P_i_S.E.
Natives	M1	460	0.057690	0.053712	0.631263	0.002959
	M2	489	-0.204386	0.052095	0.574783	0.002870
	M3	469	0.657908	0.053194	0.575660	0.002931
	M4	268	1.379232	0.070369	0.637624	0.003877
Archaeophytes	M1	55	-0.080596	0.155334	0.643438	0.008559
	M2	14	-0.740238	0.307881	0.586036	0.016964
	M3	133	0.183632	0.099890	0.574020	0.005504
	M4	10	0.672772	0.364290	0.644268	0.020072
Neophytes	M1	47	-0.470309	0.168034	0.641860	0.009259
	M2	28	-0.659093	0.217705	0.609281	0.011995
	M3	69	-0.356670	0.138683	0.586829	0.007641
	M4	12	-0.496206	0.332550	0.632806	0.018323

Supplementary Note 1: Species categorization

Lists of vascular plant species for 302 reserves in the Czech Republic were collected and updated from published records and floristic inventories at the Agency for Nature Conservation and Landscape Protection, Prague[25,56]. Species were classified as natives, archaeophytes and neophytes according to their residence time in Central Europe (following the criteria proposed by Pyšek *et al*.[26]). Archaeophytes are defined as plant species that were intentionally or unintentionally introduced into Europe between the initiation of agricultural activities during the Neolithic period (ca. BC 4000) and the European exploration of the Americas (ca. AD 1500)[9,28-29,60], respectively. Plant species introduced into Europe after AD 1500 were classified as neophytes. The two groups differ in their invasion characteristics and ecology due to the contrasting regimes of selection and cultivation operating in ancient and modern societies[29]. Most archaeophytes originated from southern Europe and most are associated with dry habitats, grasslands and agricultural landscapes, whereas most neophytes originated from outside Europe and are common in warm areas, where they invade different habitats on both dry and wet sites[27]. The separation between natives and archaeophytes in regional floras relies on a combination of paleobotanical, archaeological, ecological and historical evidence[9,60]. We tested the two hypotheses along the INIC directly using these three species assemblages.

In Fig. 6 in the main text fingerprints (barcodes) of species composition of 135 families were compared with 10,000 random draws of equal number species from the combined assemblage of all species. Families were sorted according to the number of species in the total assemblage and arranged from left to right (and from top to bottom for the inset). For modules identified for different assemblages (e.g. Fig. 1), we also compared their family composition with the families of their assemblage (10,000 non-overlapping re-samplings). Specifically, neophytes are over represented in 11 families (e.g. Fabaceae, Brassicaceae and

Amaranthaceae) but are underrepresented in Poaceae and Ranunculaceae; archaeophytes are overrepresented in 11 families (e.g. Poaceae, Brassicaceae, Lamiace and Chenopodiaceae); natives are overrepresented in 6 families (e.g. Cyperaceae, Rosaceae, Ranunculaceae and Orchidaceae), but are underrepresented in another 6 families (e.g. Brassicaceae, Chenopodiaceae and Geraniaceae). For the modules of neophytes, module 1 overrepresents Pinaceae; module 2 over represents Fabaceae and Amaranthaceae; module 3 over represents Cucurbitaceae; module 4 overrepresents Berberidaceae; module 6 overrepresents Apiaceae and Polygonaceae. For modules of archaeophytes, module 2 underrepresents Asteraceae; module 3 overrepresents Violaceceae; module 5 overrepresents Asteraceae. For modules of natives, module 1 overrepresents 8 families (e.g. Fabaceae, Caryophylaceae and Brassicaceae) but underrepresents 3 families (Rubiaceae, Juncaceae and Cyperaceae); module 2 overrepresents 9 families (e.g. Cyperaceae, Polygon, Salicaceae and Potamogaceae) but underrepresents 3 families (Asteraceae, Rosaceae and Liliaceae); module 3 overrepresents 5 families (e.g. Lamiaceae, Fagaceae and Fumariaceae) but underrepresents 1 family (Asteraceae); module 4 overrepresents 8 families (e.g. Asteraceae, Ericaceae and Juncaceae) but underrepresents 5 families (e.g. Lamiaceae, Apiaceae and Brassicaceae) (see Fig. 6).

Although there are many more native species than archaeophytes and neophytes, the modularity analysis in the main text is reliable. First, we need to emphasize that differences in the number of species between assemblages are real, and do not reflect a sampling bias. Second, if we make a crude analogy of representing reserves as resources and plants consumers, other studies showed no correlation between consumer richness and modularity for 61 real resource-consumer networks (Pearson's r = 0.18, P = 0.15; Nuwagaba, S., Zhang, F. & Hui, C. Diet choice explains the emergence of architecture in antagonistic networks. Unpublished). Finally, we did not directly compare the modularity of these three assemblages; rather, for each assemblage, we conducted null-model tests and calculated the deviation of the observed modularity to the null expectation. Consequently, we are essentially comparing how

much the modularity of each assemblage deviates from its null model expectation. Even for neophytes (156 species), the network contains 156×302 = 47112 potential connections. This number of nodes is considered a large network in literature and large enough to eliminate any potential effect of small numbers. Consequently, we think that the observation that larger networks are more modulated is a coincidence in our case, and we argue that the result of rising modularity with residence time is reliable, and not an artefact.

To justify categorizing species according to residence time and to demonstrate that no inherent differences exist between assemblages other than with respect to residence time, we demonstrate that these native species can become invasive in other regions. This means that there is no fundamental ecological difference between the components of these assemblages, which may then predispose them to successful establishment in new communities, except for the differences in residence time. Specifically, we demonstrate that a substantial portion of these natives are also invaders elsewhere in the world, and that the process of biotic homogenization has blurred the trait difference between native and introduced species. First, as North America and Europe have long been connected by trade and migration, we checked the proportion of natives in our central European list which have been naturalized in North America, using the species list of the Biota of North America Program (BONAP, www.bonap.org; personal communication with J.T. Kartesz for updated 2009 version)[61]. BONAP includes 3683 vascular plant species that occur in North America. Of the 1688 native species on our list, 486 have been introduced and naturalised in North America; that is, 28.8% of these natives have become invaders in North America.

The second dataset that we examined is Rod Randall's Global Compendium of Weeds (GCW, www.hear.org/gcw/)[62] which includes 24601 plant species that have become weedy in some parts of the world. 1162 of the 1688 native species included in our analyses appear on the GCW list - 68.8% of these natives are naturalized somewhere in the world. These two comparisons show that there is no fundamental distinction in terms of biological traits

between native and introduced species, and therefore their ability to establish in new environments. This argument is consistent with the mainstream literature in invasion biology. For instance, Dawson *et al*.[63] found that alien and native plant species behave rather similarly in response to fertilisation and competition. In a paper on perspectives and paradigms, Stohlgren and 13 leading invasion biologists[64], including two authors of this paper have argued the same point - that native and alien plant species are poorly distinguished in terms of traits. These contributions support our categorization of species only according to the residence time.

Supplementary Note 2: Modularity for the combined assemblage of all species and reserves

To support the contention that biotic interactions between natives, archaeophytes and neophytes have trivial effects on the modularity analysis at the regional scale[3,6], we analyzed the modularity for the combined assemblage of all species and reserves using the same method as detailed in the main text. Four modules were identified for the combined assemblage, with M_Z = 163.61 (P < 0.01, from 100 randomizations); this indicates significant compartmentalization. The modules that were identified were largely consistent with those identified for separate assemblages, indicating a roughly 1-to-1 matching (see Fig. 2). Specifically, modules 1, 2, 3 and 4 of all species and reserves correspond to, respectively, modules 2, 4, 1 and 3 of natives, modules 3, 6, 2 and 5 of neophytes, and modules 4, 6(species)/5(reserves), 2 and 3 of archaeophytes. This justifies the modularity analysis separately for natives, archaeophytes and neophytes in the main text.

Once modules were identified for the combined assemblage, we then calculated the within-module degree (z_i) and participation coefficient for each species (P_i)[52]. These two coefficients depict how the node in a network is positioned in its own module and with respect to other modules[53-54]. As this is a bipartite network, we slightly modified the definition of within-module degree and participation coefficient to consider only species, with species A and B considered connected by one edge if they both occur in one reserve and connected by multiple edges if they co-occur in multiple reserves. The summary of the within-module degree and participation coefficient for all modules is listed in Supplementary Table S1.

We also conducted an analysis of variance (ANOVA) for both within-module degree and participation coefficient, with assemblages and modules as factor variables. Results suggest that the within-module degree differed for assemblage (natives, archaeophytes and neophytes) ($F_{2,2042}$ = 39.04, P < 0.001) and modules (1, 2, 3 and 4 for the combined assemblage with all species) ($F_{3,2042}$ = 13.07, P < 0.001) (see below Supplementary Figure

S1A), with a strong interaction between assemblages and modules ($F_{6,2042} = 3.29$, $P = 0.003$). In contrast, the participation coefficient does not differ between assemblages ($F_{2,2042} = 2.38$, $P = 0.09$), but differed between modules ($F_{3,2042} = 47.04$, $P < 0.001$) (see Supplementary Figure S1B), with no interaction between assemblage and module ($F_{5,2042} = 0.97$, $P = 0.45$).

Supplementary Note 3: Lotka-Volterra model of a meta-community

We demonstrated in the main text that the modularity of the vascular plant assemblages in the central European reserves increases with residence time, using the list of real species grouped into neophytes (introduced after 1500AD), archaeophytes (introduced between 4000BC and 1500AD) and natives (established in Central Europe before 4000BC). To demonstrate the increase in modularity with residence time, the ideal situation would be to illustrate the network structure of the entire assemblage in a region over a long time. However, this is not feasible due to the lack of such data, since introduction and colonization events have been happening for millenniums. Consequently, we strengthened our usage of the assemblage-for-time substitution in two ways: (i) to clarify temporal dynamics of species assemblages and their connection with the three assemblage used in the main text; and (ii) to demonstrate the modularity dynamics from a mathematical model which simulates processes of competition and dispersal in a meta-community, an analogy to the vascular plants in the Central European reserves.

The temporal dynamics of the regional species assemblage can be complex. Current assemblages comprise species that have survived until now, whereas historical snapshots remove species that will go extinct over time and those added through introduction events (Supplementary Figure S3). Simply combining current observed species assemblages (e.g. natives versus natives + archaeophytes) is problematical because extinct species (dashed bars in Supplementary Figure S3) are missing in the current assemblages; the number of extinct species will increase as one goes further back in time. As illustrated in Supplementary Figure S3, the problem of, for instance, comparing native assemblage with natives plus archaeophytes is not the same as comparing the assemblages at t_4 with those at t_3 because different numbers of natives and archaeophytes have been lost and new species will still be added into the archaeophyte set. In this regard, by lumping all three assemblages together (as

in Supplementary Note 2), we only address whether the modules identified separately for each assemblage are consistent with those identified for the combined assemblage (i.e. whether modules detected for a subset assemblage is equal to modules detected for the entire assemblage). By comparing the modularity of the three assemblages (natives, archaeophytes and neophytes), we are therefore comparing survived species that undergo different time lengths of selection.

To justify the assemblage-for-time substitution, we need to demonstrate whether (i) the modules identified for the subset assemblages are consistent with those detected for the entire assemblage (supported by Supplementary Note 2); (ii) the modularity dynamics of a subset assemblage is correlated (synchronized) with that of the entire assemblage; and (iii) the modularity of the entire species assemblage increases temporally in a meta-community with competitive species in multiple interconnected sites. Using a numerical simulation we here support the last two prerequisites of the assemblage-for-time substitution. We used a widely-applied Lotka-Volterra model that depicts competitive coexistence of multiple species in multiple sets connected by dispersal:

$$\frac{dn_{ij}(t)}{dt} = r \cdot n_{ij}(t)\left(1 - \frac{\sum_{x=1}^{s} \alpha_{ix,j} n_{xj}(t)}{k_j}\right) - e \cdot n_{ij}(t) + \frac{e}{m-1}\sum_{y=1,\neq j}^{m} n_{iy} \qquad \text{(S1)}$$

where $n_{ij}(t)$ is the population size of species i in site j at time t; $\alpha_{ix,j}$ is the competitive coefficient of species x on species i in site j; k_j the carrying capacity of site j; coefficients r, e, s and m are the intrinsic population growth rate, emigration rate, total number of species in the meta-community and total number of sites in the region, respectively (they are sets to specific constants for simplicity). The competitive coefficient $\alpha_{ix,j}$ is defined as the ratio of species x's performance in site j to species i's performance in site j, p_{xj}/p_{ij}. The performance of species i in site j is defined as the bell-shaped probability density function of a normal distribution,

$$p_{ij} = \frac{1}{\sqrt{2\pi\sigma^2}} e^{-\frac{(h_j - \mu_i)}{2\sigma^2}} \tag{S2}$$

where h_j is the resource level of site j, μ_i the most suitable resource level for species i to achieve the best performance, and σ the standard deviation of species i's performance (i.e. the niche breadth). All parameters are assigned randomly in the model. We solve this model numerically using the Euler method, with the code of the above model written in *Mathematica* 8.0 (Wolfram Research Inc.) (S3).

```
s = 40; m = 40; d = 0.1; e = 0.02;
Do[{h[j] = RandomReal[{-20, 20}];
    k[j] = Random[LogNormalDistribution[6, 2]];
  }, {j, 1, m}];
Do[{μ[i] = RandomReal[{-20, 20}]; σ[i] = 10;
  }, {i, 1, s}];
Do[{p[i, j] = PDF[NormalDistribution[μ[i], σ[i]], h[j]];
  }, {i, 1, s}, {j, 1, m}];
Do[{n[i, j, 1] = Random[LogNormalDistribution[1, 1]]},
  {i, 1, s}, {j, 1, m}];
Do[{
   Do[
    {n1[i, j] = n[i, j, t] +
        d * n[i, j, t]
         (1 - (Sum[(n[x, j, t] p[x, j] / p[i, j]), {x, 1, s}]) / k[j]);},
    {i, 1, s}, {j, 1, m}];
   Do[
    {n[i, j, t + 1] =
       Max[0, (1 - e m / (m - 1)) n1[i, j] +
         e (Sum[n1[i, y], {y, 1, m}]) / (m - 1)];}, {i, 1, s}, {j, 1, m}];
  }, {t, 1, 64}];
```

(S3)

As expected, the dynamics of population size vary dramatically (Fig. 3). Some sites become unsuitable for some species, and a suite of uniquely combined species eventually

settle and persist at given sites. In contrast to the rather chaotic population dynamics, the network structure as depicted by the species-by-site matrix (Fig. 4) showed a steady trend from randomness to more ordered structures. The modularity and M_Z calculated using Netcarto for the entire assemblage (100 randomization runs) showed a steady increase, contrasting the steadiness of the null model modularity (Supplementary Figure S2A and B). Overall, the network structure evolves from counter-compartmentalization to highly modulated structure (Supplementary Figure S2B). Furthermore, when we consider only a subset of species (50% of species in Supplementary Figure S2C), the standard modularity M_Z of the subset assemblage behaves rather similarly to the entire assemblage, thus exhibiting a strong positive correlation with M_Z of the entire assemblage (Supplementary Figure S2C). This numerical demonstration suggests that the assemblage-to-time substitution is theoretically valid and provides a good way of dealing with the lack of real data to validate our assumptions.

Supplementary References:

60. Preston, C.D., Pearman, D.A. & Hall, A.R. Archaeophytes in Britain. *Bot J Linn Soc* **145**, 257-294 (2004).

61. Kartesz, J.T. & Meacham, C.A. Synthesis of the North American Flora. North Carolina Botanical Garden, Chapel Hill (1999).

62. Randall, R.P. A global compendium of weeds. Department of Agriculture and Food, Western Australia (2002).

63. Dawson, W., Fischer, M. & van Kleunen, M. Common and rare plant species respond differently to fertilisation and competition, whether they are alien or native. *Ecol Lett* **15**, 873-880 (2012).

64. Stohlgren et al. Widespread plant species: natives versus aliens in our changing world. *Biol Invasions* **13**, 1931-1944 (2011).
